# Effect of chemotherapy timing in triple-negative breast cancer: a real-world evidence study

**DOI:** 10.1007/s10549-025-07716-4

**Published:** 2025-05-21

**Authors:** Noiver Graciano, Lucelly López, Carlos A. Rodriguez, Katherine Montoya, Diego M. González, Luis Rodolfo Gómez, Maycos L. Zapata, Javier Cortés

**Affiliations:** 1 Gynecologic Oncology Unit, Instituto de Cancerología Las Américas-AUNA, Medellín, Colombia; 2 Faculty of Medicine, Universidad Pontificia Bolivariana, Medellín, Colombia; 3 Department of Pharmacology and Toxicology, Faculty of Medicine, Universidad de Antioquia, Carrera 51D # 62-29, Medellín, Colombia; 4 Information Systems Manager, Fundación AUNA Ideas, Medellín, Colombia; 5 Instituto de Cancerología Las Américas-AUNA, Medellín, Colombia; 6 Department of Internal Medicine, Faculty of Medicine, Universidad de Antioquia, Medellín, Colombia; 7 International Breast Cancer Center (IBCC), Pangaea Oncology, Quiron Group, Barcelona, Spain; 8 IOB Institute of Oncology Madrid, Hospital Beata María Ana, Madrid, Spain; 9 Oncology Department, Hospital Universitario Torrejón, Ribera Group, Madrid, Spain; 10 Faculty of Biomedical and Health Sciences, Department of Medicine, Universidad Europea de Madrid, Madrid, Spain; 11 Medica Scientia Innovation Research (MEDSIR), Barcelona, Spain

**Keywords:** Triple-negative breast cancer, Neoadjuvant chemotherapy, Adjuvant chemotherapy, Survival analysis, Event-free survival, Propensity score

## Abstract

**Purpose:**

Triple-negative breast cancer (TNBC) is an aggressive, heterogeneous malignancy with poor prognosis. The optimal timing of chemotherapy—neoadjuvant (NACT) versus adjuvant (ACT)—remains controversial. This study assessed real-world outcomes in non-metastatic TNBC patients according to chemotherapy timing.

**Methods:**

This retrospective study (2008–2023) evaluated the impact of chemotherapy timing on overall survival (OS) and event-free survival (EFS) in a cohort of 711 patients. Propensity score (PS) matching with preoperative variables was used to adjust for baseline imbalances, and Cox regression models were applied to account for treatment-related variables.

**Results:**

NACT was administered to 525 patients (73.8%), with a 37.3% pathological complete response (pCR) rate. PS matching yielded 177 patient pairs; tumor stage, age and histologic grade remained unbalanced. In the unadjusted analysis, NACT was associated with worse OS (HR 1.56, 95% CI1.08–2.25, p = 0.018). However, multivariate analysis adjusting for unmatched and postoperative variables showed a potential benefit of NACT for OS (HR 0.53, 95% CI 0.07–4.13, p = 0.545) and EFS (HR 0.94, 95% CI 0.21–4.17, p = 0.932). Tumor stage acted as an effect modifier, and stratified analyses revealed that NACT was superior to ACT in patients with advanced-stage disease who achieved pCR (HR 0.22, 95% CI 0.07–0.7, p < 0.010).

**Conclusions:**

In our TNBC cohort, chemotherapy timing significantly influenced OS and EFS, particularly in relation to initial tumor stage and pCR status. NACT was more beneficial than ACT in patients with advanced disease who achieve pCR, underscoring its role in both prognostic stratification and therapeutic decision-making.

**Supplementary Information:**

The online version contains supplementary material available at 10.1007/s10549-025-07716-4.

## Introduction

Triple-negative breast cancer (TNBC) is defined by the absence of immunohistochemical expression of hormonal receptors (HR) for estrogen and progesterone, as well as the HER2 receptor. Compared to other subtypes, TNBC is associated with a worse prognosis. Gene expression profiling of this tumor subgroup reveals heterogeneous patterns, reflecting its complex biology [1]. The treatment of early-stage TNBC is primarily based on chemotherapy (CT). Prior to the introduction of immune checkpoint inhibitors (CPI), the chemotherapy backbone for TNBC was similar to that used for HR-positive and HER2-positive breast cancers, with limited studies specifically focused on TNBC [2].

The benefit of CT in breast cancer (BC) has been historically established through studies on adjuvant chemotherapy (ACT). Initially, neoadjuvant chemotherapy (NACT) was employed with the goal of converting locally advanced disease into resectable tumors. Currently, NACT also facilitates breast-conserving surgeries, allows for the evaluation of chemosensitivity, provides prognostic information, and enables the adjustment of therapy after breast surgery [3].

Evidence regarding the interchangeability of ACT and NACT regimens is limited to controlled clinical trials (CCTs) conducted in the 1980 s and 1990 s. These studies did not evaluate, or only partially considered, the presence of HR and HER2 receptors, and thus the proportion of TNBC cases was not determined. The NSABP-B18 trial assessed the impact of chemotherapy timing (doxorubicin-cyclophosphamide, without taxanes) on overall survival (OS) and event-free survival (EFS). The study concluded that there were no differences between NACT and ACT in its 9-year follow-up [4, 5]. Similarly, the EORTC 10902 trial, which used a 4-cycle FEC regimen (5-fluorouracil, epirubicin, cyclophosphamide), found no differences in OS, EFS, or locoregional control [6]. Notably, the pCR rates in the NACT groups of these studies were only 13% and 4.3%, respectively, resembling outcomes typically observed in HR-positive populations rather than in TNBC or HER2-positive cases, where pCR rates range from 40 to 65% [7, 8].

The 2018 meta-analysis by the Early Breast Cancer Trialists’ Cooperative Group (EBCTCG) concluded that there were no differences in mortality or distant recurrence between NACT and ACT. However, a higher rate of locoregional recurrence was observed in patients who received NACT. None of the studies included in the meta-analysis were exclusive to TNBC, and a variety of CT regimens were used [9].

CCTs are essential for building scientific evidence and developing new therapies, but they have limitations in generalizing findings due to ethical, resource, and infrastructure constraints. These challenges hinder their applicability in diverse settings, highlighting the importance of real-world evidence (RWE) studies [10, 11].

Given these considerations, our study aimed to evaluate whether the timing of chemotherapy affects OS and EFS in a population of non-metastatic TNBC patients. We adjusted for confounding factors at diagnosis and treatment types, using real-world data from a prospectively collected clinical research database.

## Methods

### Study design, population, and data collection

A retrospective cohort study was conducted, including all women with a diagnosis of de novo non-metastatic TNBC who underwent surgery and received at least one cycle of chemotherapy at the Instituto de Cancerología, Clínica Las Américas—AUNA (IDCLA-AUNA) in Medellín, Colombia, between January 2008 and December 31, 2023. Clinical staging was determined according to the 8 th edition of the American Joint Committee on Cancer (AJCC) criteria. TNBC status was confirmed by institutional pathologists, defined by the absence of estrogen, progesterone, and HER2 receptors. Receptor status was determined by immunohistochemistry (IHC), with a positivity threshold of ≥ 1%. HER2 status was also assessed by IHC, with a score of 2 + or 3 + confirmed by fluorescence in situ hybridization (FISH).

Patients with incomplete clinical or histopathological data, synchronous or metachronous breast cancer, metastatic disease diagnosed within three months of breast surgery, a history of other cancers (except basal or squamous cell skin cancers), or those who did not provide informed consent for the use of their medical records in research were excluded.

Demographic and clinical characteristics collected included age, social security status, history of diabetes, hypertension, parity, menopausal status, family history of breast cancer, and body mass index (BMI). Tumor-related variables included imaging modality used for diagnosis, histological subtype, histological grade (HG), and lymphovascular invasion (LVI). Histological grade was assessed using the Scarff-Bloom-Richardson criteria, while histological subtype and LVI, were evaluated according to the World Health Organization and the College of American Pathologists guidelines [12].

The decision for upfront surgery (without prior neoadjuvant therapy) and the type of surgery (mastectomy vs. breast-conserving surgery) was made by the treating breast surgeon, prioritizing breast-conserving surgery with negative margins. Chemotherapy regimens primarily included anthracyclines, cyclophosphamide, taxanes, and carboplatin. In cases where anthracyclines were contraindicated, combinations with taxanes or CMF (cyclophosphamide, methotrexate, 5-fluorouracil) were used. Chemotherapy duration was up to six months or until grade 3 toxicity unresponsive to dose adjustments or grade 4 toxicity, as defined by the Common Terminology Criteria for Adverse Events (CTCAE) versions 4.0 or 5.0. Radiotherapy (RT) was indicated by radiation oncologists based on breast-conserving surgery, positive margins, nodal involvement, or tumor size > 5 cm in mastectomy cases. RT was administered after ACT. Controversial cases regarding surgery type, chemotherapy, or RT were discussed in the institutional multidisciplinary breast cancer group.

Data were extracted from the Institutional Registry, Breast Cancer Module, recorded in a RedCap database, which includes all clinical records of patients treated at the institution. Any inconsistencies or missing data identified by the investigators were verified in the IDCLA medical records and corrected or completed for analysis. The study was approved and audited by the Institutional Ethics Committee (Act 220–2024) and adhered to international and national ethical standards (Declaration of Helsinki and Resolution 8430 of 1993 from Colombia Ministry of Health).

### Exposures and outcomes

The primary exposure variable in this study was the timing of chemotherapy, specifically comparing NACT with ACT. To adjust the analysis, several independent variables were considered. These included factors present at diagnosis (first or second contact with an IDCLA specialist), such as social security status (SS), age, family history of breast cancer, clinical tumor stage, BMI, HG, and LVI.

Additionally, postoperative variables such as pCR, radiotherapy, the type of surgery performed, the chemotherapy regimen (classified as anthracycline-based versus non-anthracycline-based), receipt of three or more chemotherapy cycles were analyzed.

Outcome variables were defined according to STEEP 2.0 (Standardized Definitions for Efficacy End Points in Adjuvant Breast Cancer Clinical Trials version 2.0) [13] and NeoSTEEP (Standardized Definitions for Efficacy End Points in Neoadjuvant Breast Cancer Clinical Trials) [14] systems. The primary outcome, overall survival (OS), was operationalized as the time from curative-intent surgery until death from any cause or the last institutional contact with the patient, with death dates verified through the National Civil Registry up to April 30, 2024. The secondary outcome, event-free survival (EFS), was defined as the time from curative-intent surgery (ACT group) or from the first dose of chemotherapy (NACT group) to local recurrence, distant recurrence, contralateral breast cancer, death from any cause, or the last institutional contact.

### Data analysis

To describe the study population, summary measures were calculated. For clinical variables and follow-up intervals, measures of central tendency and dispersion were used, depending on the results of the Shapiro–Wilk normality test, utilizing mean and standard deviation or median and interquartile range as appropriate. Qualitative variables, such as HG, LVI, and stage, were presented as absolute frequencies and percentages.

The analysis focused on patients with TNBC who received chemotherapy and underwent surgery, using the timing of chemotherapy as the primary exposure variable. A PS with preoperative variables was estimated using the nearest neighbor method with a caliper of 0.1, determining the probability of patients being classified into the primary exposure group. This probability was calculated based on the variables assessed at diagnosis: SS, age, family history of breast cancer, clinical tumor stage, BMI, HG, and LVI.

Kaplan–Meier survival curves were constructed to analyze OS and EFS, while the Log-Rank test was used to evaluate the association between chemotherapy timing and these outcomes. A Cox proportional hazards model was applied, adjusting for unadjusted variables after PS showing significant differences (p < 0.1), radiotherapy use, chemotherapy timing, chemotherapy type, and whether patients received three or more chemotherapy cycles, thereby estimating HR with corresponding confidence intervals. The proportional hazards assumption was verified, with a p-value of less than 0.05 considered statistically significant.

Given the importance of tumor stage and pCR in the effect of chemotherapy timing (especially NACT) on the evaluated outcomes, a stratified analysis was performed based on pCR status compared to ACT. Additionally, a sensitivity analysis was conducted that included only stage I and II patients, as well as those classified as cT1-2, cN0 in the NACT group and pT1-2, pN0 in the ACT group.

Statistical analyses were performed using R version 4.4.2 and Jamovi version 2.6.17, utilizing R packages such as Matchlt, Matching, Cobalt, and Psych for propensity score calculations.

## Results

### Clinical and tumor characteristics at diagnosis and treatment

**Fig. 1:**
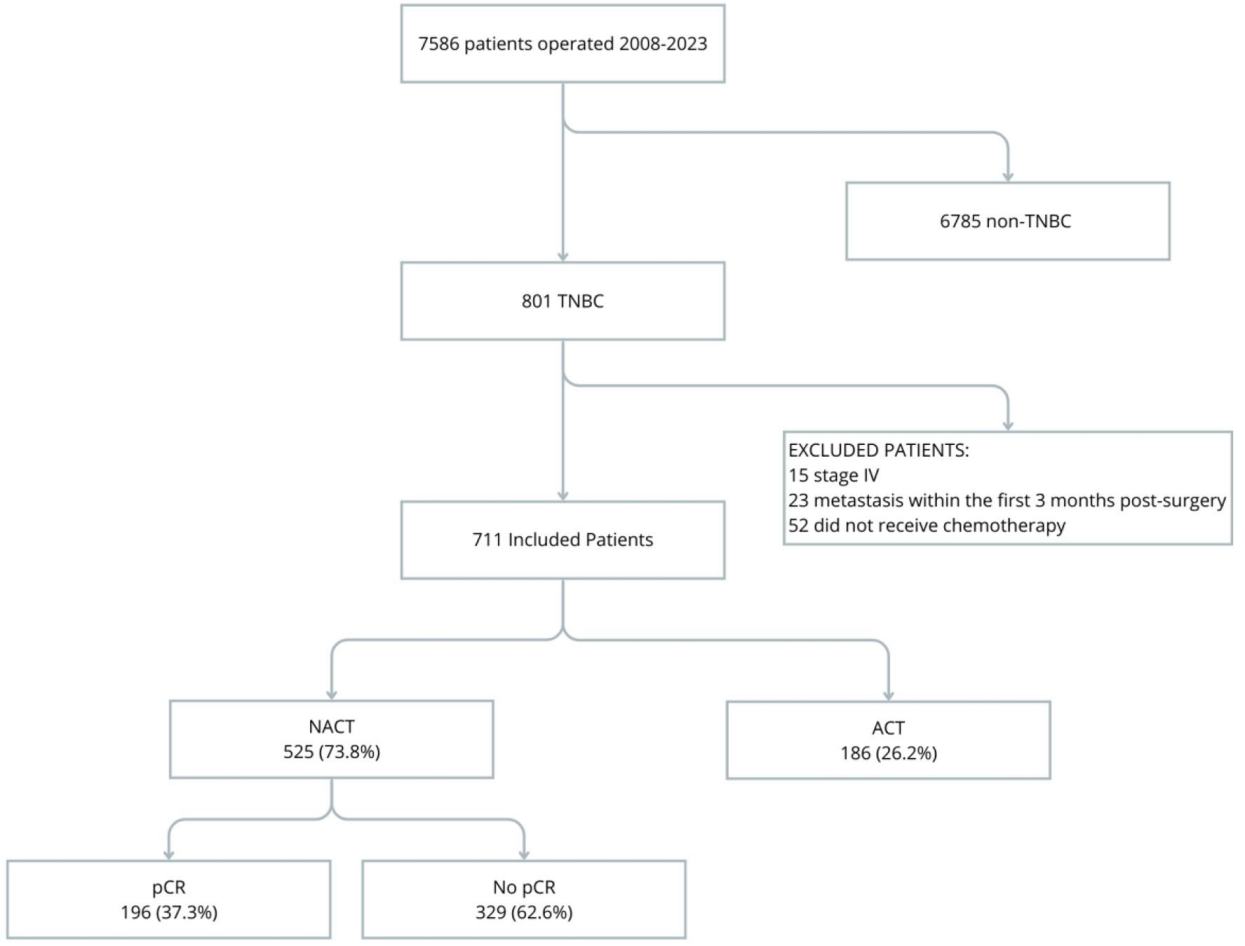
Flowchart of patient selection from 2008 to 2023 showing the timing of chemotherapy (NACT vs ACT) and the pathological complete response (pCR) to neoadjuvant chemotherapy Flowchart of patient selection

During the study period (2008–2023), 7,586 patients with non-metastatic breast cancer were identified, of whom 811 (10.7%) had TNBC, and 711 met the eligibility criteria. Among these, 73.8% received NACT, achieving a pathological complete response (pCR) rate of 37.3% (Fig. 1). Baseline characteristics of the entire cohort, as well as the NACT and ACT groups, are presented in Table 1. The median age of the cohort was 54 years (IQR 45–63), with 36% having a family history of breast cancer, 66% being postmenopausal, 83% having children, and 63% presenting with overweight or obesity (41% and 22%, respectively).

**Table 1:** Characteristics of patients at diagnosis of TNBC according to chemotherapy and surgery

Variable	Total N = 711	ACT, N = 186	NACT, N = 525	P value	ACT N = 177	NACT, N = 177	P Value
		Unmatched cohort			PS-matched cohort		
Age in years, median (IQR)	54 (45, 63)	59(49, 68)	52 (44, 61)		59 (49, 68)	50 (43, 60)	
Categorized age in years				< 0.01			< 0.01
< 35	53 (7.5%)	6 (3.2%)	47 (9.0%)		6 (3.4%)	18 (10%)	
35–50	237 (33%)	43 (23%)	194 (37%)		42 (24%)	73 (41%)	
51–70	348 (49%)	99 (53%)	249 (47%)		92 (52%)	77 (44%)	
> 70	73 (10%)	38 (20%)	35 (6.7%)		37 (21%)	9 (5.1%)	
Social insurance				0.45			0.30
Contributory	630 (89%)	162 (87%)	468 (89%)		154 (87%)	147 (83%)	
Subsidized	81 (11%)	24 (13%)	57 (11%)		23 (13%)	30 (17%)	
Familial breast cancer				0.08			1.00
No	452 (64%)	128 (69%)	324 (62%)		120 (68%)	120 (68%)	
Yes	259 (36%)	58 (31%)	201 (38%)		57 (32%)	57 (32%)	
Menopause state				< 0.01			0.24
Menopause	468 (66%)	140 (75%)	328 (62%)		131 (74%)	121 (68%)	
Premenopause	243 (34%)	46 (25%)	197 (38%)		46 (26%)	56 (32%)	
Diabetes mellitus (DM)				0.33			0.31
No	654 (92%)	168 (90%)	486 (93%)		160 (90%)	154 (87%)	
Yes	57 (8.0%)	18 (9.7%)	39 (7.4%)		17 (9.6%)	23 (13%)	
Hypertension (HTN)				0.31			0.49
No	491 (69%)	134 (72%)	357 (68%)		125 (71%)	119 (67%)	
Yes	220 (31%)	52 (28%)	168 (32%)		52 (29%)	58 (33%)	
Children				0.27			0.21
Yes	589 (83%)	159 (85%)	430 (82%)		150 (85%)	141 (80%)	
No	122 (17%)	27 (15%)	95 (18%)		27 (15%)	36 (20%)	
Body Mass Index (BMI)				0.37			0.54
Normal	260 (37%)	76 (41%)	184 (35%)		71 (40%)	77 (44%)	
Overweight	293 (41%)	71 (38%)	222 (42%)		69 (39%)	71 (40%)	
Obese	158 (22%)	39 (21%)	119 (23%)		37 (21%)	29 (16%)	
Imaging				0.03			0.74
Mammography	500 (70%)	119 (64%)	381 (73%)		114 (64%)	117 (66%)	
Ultrasound or MRI	211 (30%)	67 (36%)	144 (27%)		63 (36%)	60 (34%)	
Histology				0.07			0.53
Ductal	640 (90%)	161 (87%)	479 (91%)		156 (88%)	152 (86%)	
Non-ductal	71 (10.0%)	25 (13%)	46 (8.8%)		21 (12%)	25 (14%)	
Histologic grade (HG)				0.05			< 0.01
1	19 (2.7%)	6 (3.2%)	13 (2.5%)		5 (2.8%)	8 (4.5%)	
2	125 (18%)	22 (12%)	103 (20%)		18 (10%)	40 (23%)	
3	567 (80%)	158 (85%)	409 (78%)		154 (87%)	129 (73%)	
Lymphovascular invasion (LVI)				0.05			0.10
No	618 (87%)	49 (31%)	47 (9.8%)		150 (85%)	138 (78%)	
Yes	93 (13%)	108 (69%)	432 (90%)		27 (15%)	39 (22%)	
Tumor Stage				< 0.01			< 0.01
I	122 (17%)	83 (45%)	39 (7.4%)		76 (43%)	11 (6.2%)	
II	358 (50%)	89 (48%)	269 (51%)		87 (49%)	90 (51%)	
III	231 (32%)	14 (7.5%)	217 (41%)		14 (7.9%)	76 (43%)	
Clinical T category							
cT0	1 (0.1%)	1 (0.5%)	0 (0%)				
cT1	145 (20%)	91 (49%)	54 (10%)				
cT2	308 (43%)	69 (37%)	239 (46%)				
cT3	145 (20%)	15 (8.1%)	130 (25%)				
cT4	112 (16%)	10 (5.4%)	102 (19%)				
Clinical N category							
cN0	383 (54%)	148 (80%)	235 (45%)				
cN1	227 (32%)	33 (18%)	194 (37%)				
cN2	86 (12%)	4 (2.2%)	82 (16%)				
cN3	15 (2.1%)	1 (0.5%)	14 (2.7%)				

*TNBC* Triple negative breast cancer, *NACT* Neoadjuvant chemotherapy, *ACT* Adjuvant chemotherapy, *IQR* Interquartile range, *MRI* Magnetic resonance imaging

Compared to the ACT group, the NACT group included more patients aged ≤ 50 years (46% vs. 26.2%), premenopausal women (38% vs. 25%), and those with stage III tumors at diagnosis (41% vs. 7.5%), nodal involvement (55% vs. 20%), T3-4 tumors (44% vs. 13.5%), and lymphovascular invasion (LVI) (90% vs. 69%).

In the entire cohort, most patients received RT (70%), anthracycline-based chemotherapy (66%), and ≥ 3 cycles of chemotherapy (96%). Surgical approaches were balanced between mastectomy (50%) and breast-conserving surgery (50%). Compared to NACT, the ACT group had a higher rate of breast-conserving surgery (60% vs. 46%), greater use of anthracyclines (73% vs. 64%), and lower use of RT (65% vs. 72%). Carboplatin (CBP) was only used in the NACT group in 36% of patients (Table 2).

**Table 2:** Treatment variables and responses

Variable	Total N = 711	ACT, N = 186	NACT, N = 525
Pathological T category			
pT0	220 (31%)	2 (1.1%)	218 (42%)
pT1	255 (36%)	89 (48%)	166 (32%)
pT2	153 (22%)	69 (37%)	84 (16%)
pT3	59 (8.3%)	22 (12%)	37 (7.0%)
pT4	24 (3.4%)	4 (2.2%)	20 (3.8%)
Pathological N category			
pN0	478 (67%)	116 (62%)	362 (69%)
pN1	145 (20%)	47 (25%)	98 (19%)
pN2	56 (7.9%)	12 (6.5%)	44 (8.4%)
pN3	32 (4.5%)	11 (5.9%)	21 (4.0%)
Type of Surgery			
Conservative	352 (50%)	111 (60%)	241 (46%)
Mastectomy	359 (50%)	75 (40%)	284 (54%)
Radiotherapy			
Yes	499 (70%)	120 (65%)	379 (72%)
No	212 (30%)	66 (35%)	146 (28%)
Anthracyclines			
Yes	470 (66%)	135 (73%)	335 (64%)
No	241 (34%)	51 (27%)	190 (36%)
< 3 Cycles of CT			
No	684 (96%)	169 (91%)	515 (98%)
Yes	27 (3.8%)	17 (9.1%)	10 (1.9%)
Carboplatin			
Yes	187 (36%)	NA	187 (36%)
No	338 (64%)	NA	338 (64%)
pCR			
Yes	196 (37%)	NA	196 (37%)
No	329 (63%)	NA	329 (63%)

*NACT* neoadjuvant chemotherapy, *ACT* adjuvant chemotherapy, *pCR* pathologic complete response, *CT* Chemotherapy

### Clinical outcomes

Before PS matching, the median follow-up for the entire cohort was 43 months (IQR 23.4–75.3), with all-cause mortality rate of 29.7% and a recurrence rate of 20.5%. In the unadjusted analysis, ACT was associated with longer overall survival (OS) (median 154 vs. 139 months) and EFS (median 154 vs. 129 months), although the differences were not statistically significant (OS: HR 1.30, 95% CI 0.95–1.77, p = 0.13; EFS: HR 1.18, 95% CI 0.88–1.58, p = 0.274). The 3-, 5-, and 10-year OS rates for the NACT vs. ACT groups were 80%, 66.5%, and 53.5% vs. 86.2%, 72.6%, and 61.4%, respectively. Similarly, the 3-, 5-, and 10-year EFS rates were 72.3%, 62.9%, and 51.8% vs. 76.2%, 65.4%, and 55.0%, respectively.

**Fig. 2:**
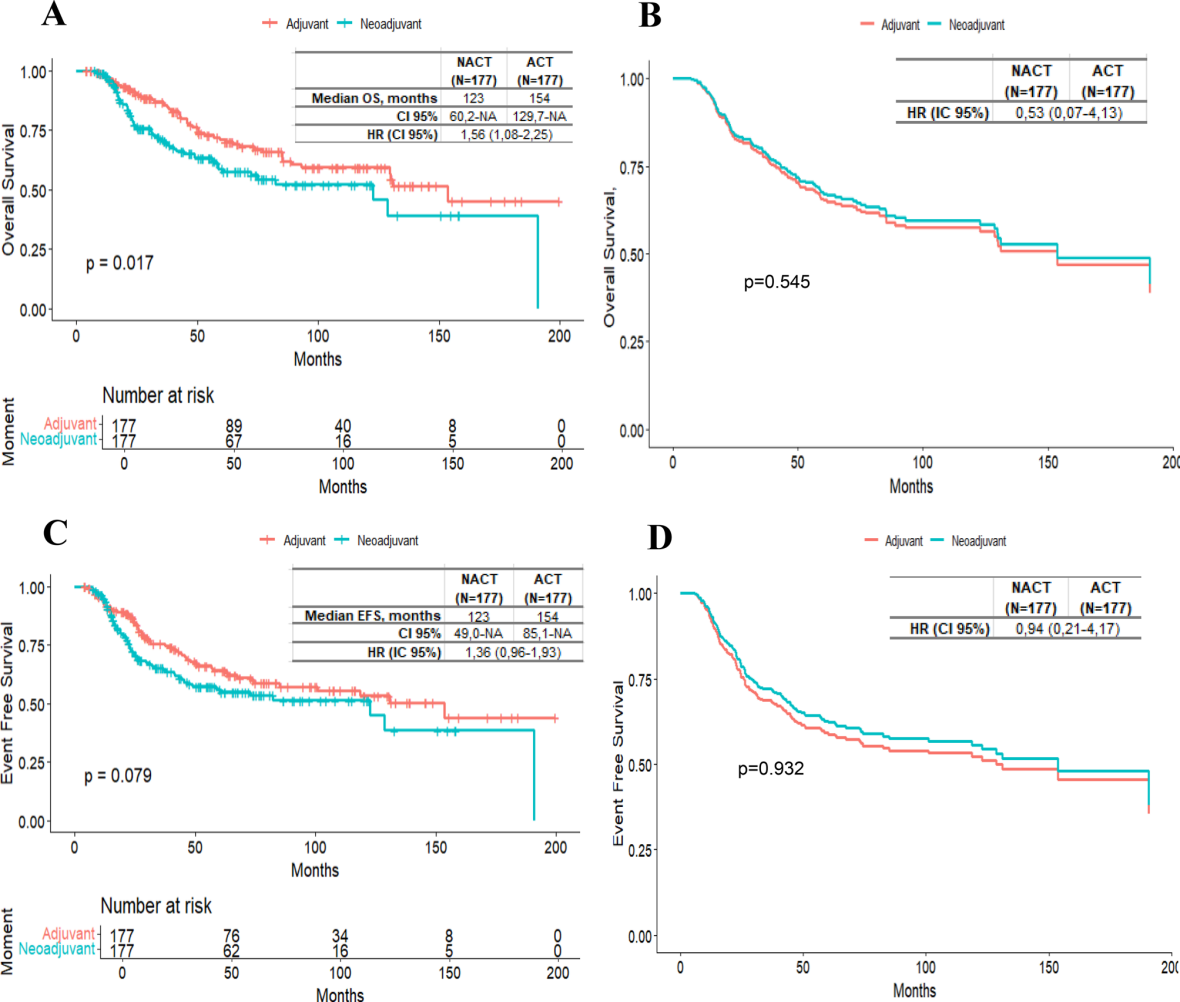
Survival outcomes with NACT vs ACT in the PS-matched cohort of TNBC patients. Overall survival (OS) and event-free survival (EFS) in TNBC patients treated with NACT vs ACT in PS-matched cohort. **A**: unadjusted OS. **B**: Adjusted OS for variables considered in Model 2 using Cox regression. **C**: Unadjusted EFS. **D**: Adjusted EFS for variables considered in Model 2 using Cox regression

After PS matching, most variables were balanced, generating 177 matched pairs. However, age, tumor stage, and histological grade (HG) remained unbalanced. In the adjusted cohort, NACT was associated with an increased risk of mortality and recurrence (OS: HR 1.56, 95% CI 1.08–2.25, p = 0.018; EFS: HR 1.36, 95% CI 0.96–1.93, p = 0.08) (Table 3, Fig. 2).

**Table 3:** Overall Survival (OS) and Event-Free Survival (EFS) in TNBC Patients Treated with NACT versus ACT in a PS-Matched Cohort using cox proportional hazards analysis

**Overall Survival (OS)**				**Model 1**			**Model 2**		
Variable	Crude HR	95% CI	P Value	Adjusted HR	95% CI	P Value	Adjusted HR	95% CI	P Value
NACT vs ACT	1.56	1.08–2.25	0.018	0.99	0.62–1.61	0.98	0.53	0.07–4.13	0.545
Stage II vs Stage I	2.06	1.14–3.74	0.017	1.65	0.88–3.10	0.121	1.42	0.72–2.83	0.315
Stage III vs Stage I	5.17	2.86–9.33	< 0.001	3.92	1.90–8.10	< 0.001	4.44	1.74–11.32	0.002
HG2 vs HG1	1.04	0.35–3.13	0.941	1.34	0.44–4.11	0.609	1.38	0.45–4.24	0.575
HG3 vs HG1	1.18	0.43–3.22	0.744	1.65	0.58–4.66	0.345	1.68	0.60–4.74	0.327
Age 35-50y vs < 35y	1.26	0.49–3.23	0.635	1.27	0.49–3.32	0.62	1.32	0.51–3.46	0.567
Age 51-70y vs < 35y	1.5	0.60–3.74	0.383	1.6	0.63–4.10	0.326	1.67	0.65–4.30	0.287
Age > 70y vs < 35y	2.23	0.84–5.92	0.107	2.14	0.77–6.01	0.146	2.14	0.75–6.11	0.153
No RT vs RT	1.89	1.31–2.73	0.001	1.81	1.23–2.67	0.002	1.87	1.26–2.76	0.002
< 3 CT vs ≥ 3 CT	1.61	0.84–3.09	0.148	1.51	0.74–3.07	0.259	1.56	0.76–3.19	0.222
Non-Anthracyclines vs Anthracyclines	0.88	0.57–1.38	0.587	0.8	0.48–1.33	0.385	0.78	0.47–1.29	0.328
Moment_NACT* Stage II							2.28	0.27–19.34	0.449
Moment_NACT* Stage III							1.49	0.16–13.81	0.723

**Event-Free Survival (EFS)**				**Model 1**			**Model 2**		
Variable	Crude HR	95% CI	P Value	Adjusted HR	95% CI	P Value	Adjusted HR	95% CI	P Value
NACT vs ACT	1.36	0.96–1.93	0.08	0.89	0.57–1.40	0.613	0.94	0.21–4.17	0.932
Stage II vs Stage I	2	1.15–3.49	0.014	1.69	0.93–3.08	0.083	1.6	0.83–3.10	0.159
Stage III vs Stage I	4.65	2.67–8.11	< 0.001	3.9	1.95–7.79	< 0.001	4.94	1.99–12.23	0.001
HG2 vs HG1	0.97	0.32–2.91	0.956	1.28	0.42–3.92	0.661	1.31	0.43–4.00	0.639
HG3 vs HG1	1.37	0.50–3.71	0.54	1.88	0.67–5.23	0.229	1.91	0.68–5.33	0.217
Age 35-50y vs < 35y	1.27	0.53–3.01	0.589	1.26	0.52–3.03	0.608	1.29	0.53–3.11	0.57
Age 51-70y vs < 35y	1.54	0.67–3.55	0.314	1.62	0.69–3.83	0.272	1.66	0.70–3.95	0.248
Age > 70y vs < 35y	2.06	0.83–5.11	0.119	1.85	0.71–4.83	0.211	1.74	0.65–4.65	0.27
No RT vs RT	1.79	1.26–2.53	0.001	1.71	1.19–2.47	0.004	1.75	1.21–2.53	0.003
< 3 CT vs ≥ 3 CT	1.35	0.71–2.58	0.358	1.26	0.63–2.52	0.516	1.28	0.64–2.56	0.49
Non-Anthracyclines vs Anthracyclines	0.78	0.51–1.20	0.263	0.79	0.50–1.27	0.328	0.77	0.48–1.24	0.283
Moment_NACT* Stage II							1.08	0.22–5.33	0.921
Moment_NACT* Stage III							0.72	0.13–3.94	0.704

*TNBC* Triple negative breast cancer, *NACT* Neoadjuvant chemotherapy, *ACT* Adjuvant chemotherapy, *CI* Confidence interval, *HG* Histologic grade, *RT* Radiotherapy, *CT* Chemotherapy cycles, *y* years, *HR* Hazard ratio. The type of surgery was taken into account in the model as a stratification variable

### Multivariate analysis with propensity score matching

Using the PS-matched cohort of 354 patients, a multivariate analysis was performed, including variables not controlled by PS and treatment-related variables. In this analysis, the timing of chemotherapy did not statistically affect OS (HR 0.99, 95% CI 0.62–1.61, p = 0.098) or EFS (HR 0.89, 95% CI 0.57–1.40, p = 0.613) (Model 1 in Table 3).

The variable with the greatest influence on this reversal was tumor stage, which enhanced the positive effect of NACT on OS and EFS when included as an interaction variable (OS: HR 0.53, 95% CI 0.07–4.13, p = 0.545; EFS: HR 0.94, 95% CI 0.21–4.17, p = 0.932). Conversely, patients who did not receive RT experienced significantly worse OS and EFS (OS: HR 1.87, 95% CI 1.26–2.76, p = 0.002; EFS: HR 1.75, 95% CI 1.21–2.53, p = 0.003) (Model 2 in Table 3).

### Effect of chemotherapy timing on pathological complete response and tumor stage

**Fig. 3:**
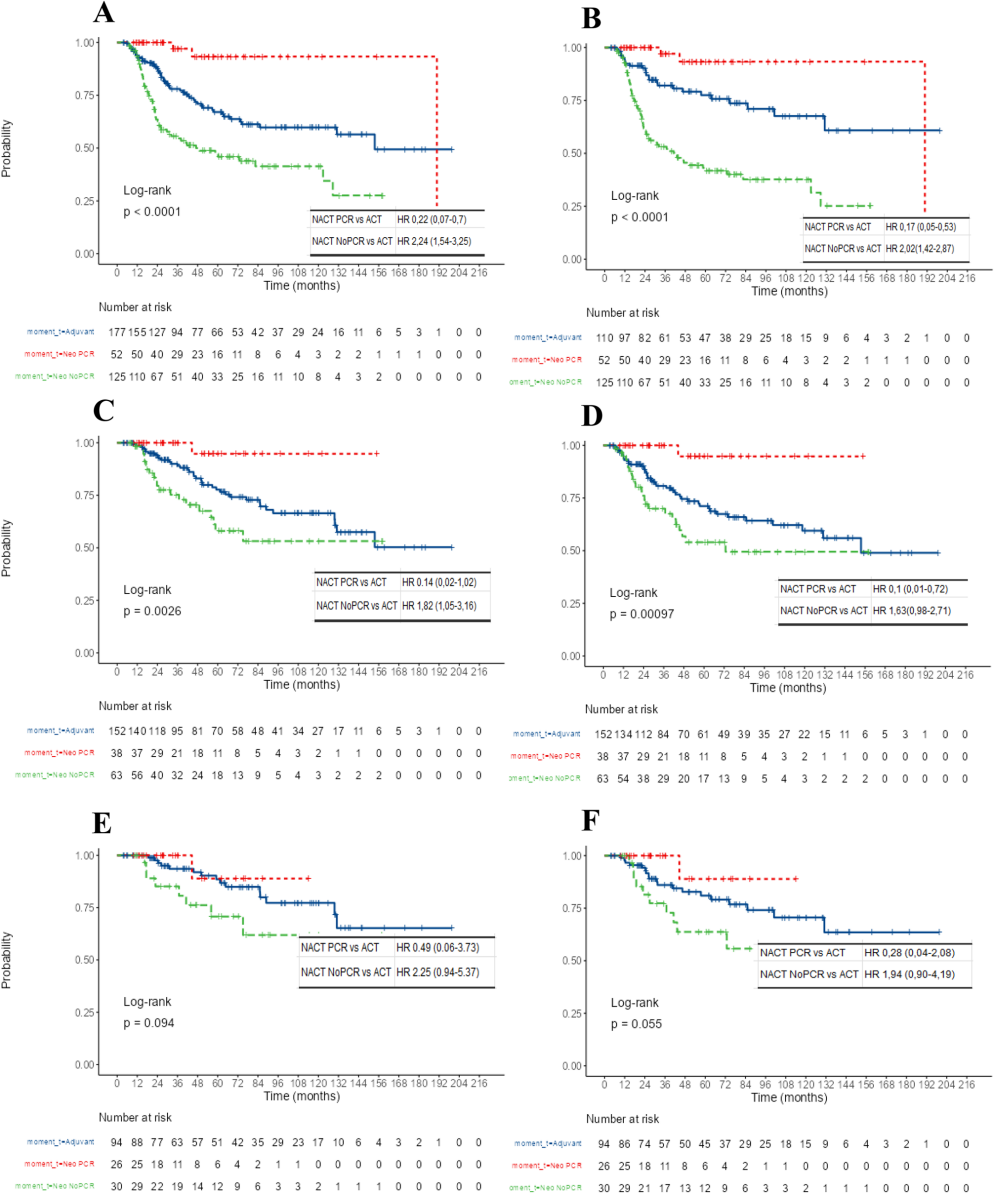
Survival outcomes with NACT vs ACT according to pathological findings and tumor stage. Comparison of overall survival OS **A** and event-free survival EFS **B** for all PS-matched cohorts receiving NACT vs ACT based on pathological findings at definitive surgery. Comparison of OS **C** and EFS **D** for tumor stages I and II. Comparison of OS **E** and EFS **F** fo cT1-2, cN0 (NACT group) and pT1-2, pN0 (ACT group). HR: hazard ratio, NACT: neoadjuvant chemotherapy, ACT: adjuvant chemotherapy

In the PS-matched cohort, the effect of NACT vs. ACT was analyzed based on pCR status. Patients who achieved pCR with NACT had significantly improved OS (HR 0.22, 95% CI 0.07–0.7) and EFS (HR 0.17, 95% CI 0.05–0.53). Conversely, patients who did not achieve pCR had worse OS (HR 2.24, 95% CI 1.54–3.25) and EFS (HR 2.02, 95% CI 1.42–2.87) (Fig. 3A and B).

A sensitivity analysis in patients with stage I and II disease showed similar results, with improved OS and EFS in those achieving pCR (OS: HR 0.14, 95% CI 0.02–1.02; EFS: HR 0.1, 95% CI 0.01–0.72) and worse outcomes in those without pCR (OS: HR 1.82, 95% CI 1.05–3.16; EFS: HR 1.63, 95% CI 0.98–2.71) (Fig. 3C and D). A similar, though less pronounced, pattern was observed in early-stage TNBC without nodal involvement (cT1-2, cN0 for NACT; pT1-2, pN0 for ACT) (Fig. 3E and F).

### Effect of carboplatin (CBP) on OS and EFS

Considering that CBP was used exclusively in the NACT group, mainly during the last seven years of the study period (2017–2023) in 36% of patients (187/525), and that after adjusting for the propensity score (PS), only 50 patients had received CBP, it was not deemed appropriate to include it as a covariate in the multivariate analysis. Nevertheless, an exploratory multivariate analysis incorporating CBP (Table S1, Supplementary Information) showed no change in the effect of chemotherapy timing on overall survival (HR 0.56, 95% CI 0.070–4.417, p = 0.578), or event-free survival (HR 0.85, 95% CI 0.185–3.878, p = 0.831), compared to the original model without CBP (Table 2).

In the overall cohort, CBP use was associated with improved OS, though the result was not statistically significant (HR 0.88, 95% CI 0.35–2.16, p = 0.772), likely due to limited sample size. Similarly, in the stratified analysis, CBP use in the NACT group showed a non-significant trend toward better OS and EFS compared with ACT (OS: HR 0.90, 95% CI 0.41–2.00, p = 0.794; EFS, HR 0.93, 95% CI 0.47–1.82, p = 0.824) (Figures S1 and S2, Supplementary Information).

## Discussion

Evidence on TNBC has been developed over the past two decades, with much of the current treatment for this aggressive breast cancer subtype extrapolated from other histological subtypes [15, 16].

In our cohort, TNBC accounted for 10.7% of cases, with two-thirds of patients being postmenopausal, overweight or obese, and presenting with stage I or II tumors. Compere with other studies which report higher TNBC prevalence, a greater proportion of premenopausal patients, and more cases of stage III disease [17, 18]. Achieving a pCR is a strong prognostic factor, and the benefit of additional interventions is limited in these cases [19, 20]. The pCR rate in our cohort was 37.3%, significantly lower than the rates reported in clinical trials (> 50%), likely due to the limited use of carboplatin (36%), anthracyclines (66%), and the lack of immunotherapy (0%) [8, 21]. However, it is consistent with the pCR rates observed in other real-world studies where immunotherapy was also unavailable [17, 18]. This aligns with the poor prognosis observed in this TNBC population, reflected by a mortality rate of 29.7%, underscoring the aggressive nature of TNBC compared to other breast cancer subtypes.

Given the prognostic impact of pCR on OS and EFS in NACT, a stratified analysis revealed that NACT was superior to ACT in patients achieving pCR but inferior in those who did not, consistent with previous studies [22–24]. Sensitivity analysis confirmed this pattern in stage I-II patients but not in those with node-negative, small tumors (T1-2), aligning with other findings [23].

The inclusion of non-TNBC subtypes, metastatic stages, and the lack of treatment-specific outcomes in many observational studies contribute to controversial findings in non-metastatic TNBC [25–27] Additionally, the absence of specific research questions and inadequate control of confounders and biases are common issues, leading to inconclusive evidence regarding the timing of chemotherapy in breast cancer [28–30]. To address these challenges, our study aimed to determine whether chemotherapy timing affects OS and EFS in non-metastatic TNBC. PS matching was used to control for key confounding variables at diagnosis that influence the selection of NACT or ACT. Despite this effort, differences in tumor stage, age, and histological grade (HG) persisted in the PS-matched cohort, however, to control the confusion generated by these variables, they were included in the Cox models. Multivariate analysis identified tumor stage at diagnosis as a key factor influencing the likelihood of receiving NACT versus ACT, as well as OS and EFS. Stage III disease increased mortality risk by 4.44 times compared to stage I and modified the effect of NACT versus ACT, acting as an interaction variable (adjusted HR changed from 1.56 to 0.53, though not statistically significant).

These findings underscore the importance of identifying clinical factors at diagnosis that predict the likelihood of achieving pCR in NACT candidates. Additionally, the role of neoadjuvant therapy in small, node-negative tumors should be carefully evaluated, as ACT offers comparable outcomes to NACT and may avoid overtreatment with current NACT regimens, which often include up to five drugs [8].

Our study has several limitations. Despite efforts to control selection bias using multiple variables in the PS model, the retrospective, single-center design and unmeasured variables (e.g., Ki67%, functional class, comorbidities, tumor-infitrating lymphocytes, and non-ductal histology) resulted in residual imbalances. PS models also carry a risk of overfitting, which, although reduced by logistic regression in matching, remains a concern. It is recommended not to exceed 10 adjustment variables per recorded event; in our case, 13 of 18 possible variables were included [31]. The median follow-up of 43 months may be short for assessing OS outcomes, although most TNBC-related deaths occur within the first three years, and the limited efficacy of treatments for recurrent disease makes this follow-up sufficient to identify high-risk, high-mortality populations [32]. Additionally, adjuvant treatments in the NACT cohort, such as capecitabine, were not recorded (mainly because it was used only in the last 3 years of the cohort and in a small number of patients). Furthermore, as immunotherapy and PARP inhibitors were unavailable for early-stage TNBC during the study period, the external validity of our findings in relation to current treatment standards may be limited. However, the findings remain highly valuable in setting where access to these therapies is unavailable, and anthracyclines and taxanes are the main treatments for patients with TNBC. Moreover, adding immune checkpoint inhibitors or the possibility of tailoring adjuvant chemotherapy with capecitabine if no pCR, might increase the benefit of NACT compared with ACT.

The role of NACT in locally advanced TNBC is reinforced in this study, not only for achieving breast-conserving surgery but also because it allows the identification of high-risk populations that require treatment intensification after surgery. Similarly, the importance of identifying patients with non-pCR who, at diagnosis, present with nodal involvement and T3 tumor sizes becomes evident. These patients exhibit behavior like high-risk TNBC populations and benefit from additional treatments in the adjuvant setting. It is also crucial to determine which other variables at the time of diagnosis are associated with a higher risk of non-pCR.

On the other hand, the benefit of NACT in patients without nodal involvement and tumor sizes ≤ 5 cm remains unclear. In such cases, ACT may still play a role in scenarios where there are challenges in administering NACT. To address this, further studies with larger sample sizes are needed to balance confounding factors such as frailty, age, or comorbidities, which may introduce selection bias in patients who are not offered NACT and are instead taken directly to surgery.

## Conclusions

Chemotherapy timing in TNBC significantly influenced survival outcomes, particularly in relation to tumor stage and pCR status. NACT was more beneficial than ACT in patients with higher tumor stage who achieve pCR, underscoring its role in both prognostic stratification and therapeutic decision-making.

## Supplementary Information

Below is the link to the electronic supplementary material.

Supplementary file1 (PDF 159 KB)

## Data Availability

The datasets generated or analyzed during the current study are not publicly available due to the institutional policies of Instituto de Cancerología Las Américas-AUNA, but are available from the corresponding author on reasonable request.

## References

[1] Leon-Ferre RA, Goetz MP (2023) Advances in systemic therapies for triple negative breast cancer. BMJ 381:e071674 37253507 10.1136/bmj-2022-071674

[2] Loibl S, André F, Bachelot T, Barrios CH, Bergh J, Burstein HJ et al (2024) Early breast cancer: ESMO clinical practice guideline for diagnosis, treatment and follow-up. Ann Oncol 35:159–182 38101773 10.1016/j.annonc.2023.11.016

[3] Agostinetto E, Gligorov J, Piccart M (2022) Systemic therapy for early-stage breast cancer: learning from the past to build the future. Nat Rev Clin Oncol 19:763–774 36253451 10.1038/s41571-022-00687-1 PMC9575647

[4] Wolmark N, Wang J, Mamounas E, Bryant J, Fisher B (2001) Preoperative chemotherapy in patients with operable breast cancer: nine-year results from national surgical adjuvant breast and bowel project B-18. J Natl Cancer Inst Monogr. 2001:96–102 10.1093/oxfordjournals.jncimonographs.a003469 11773300

[5] Fisher B, Bryant J, Wolmark N, Mamounas E, Brown A, Fisher ER et al (1998) Effect of preoperative chemotherapy on the outcome of women with operable breast cancer. JCO 16:2672–2685 10.1200/JCO.1998.16.8.2672 9704717

[6] van der Hage JA, van de Velde CJH, Julien J-P, Tubiana-Hulin M, Vandervelden C, Duchateau L et al (2001) Preoperative chemotherapy in primary operable breast cancer: results from the European organization for research and treatment of cancer trial 10902. JCO 19:4224–4237 10.1200/JCO.2001.19.22.4224 11709566

[7] Schneeweiss A, Chia S, Hickish T, Harvey V, Eniu A, Hegg R et al (2013) Pertuzumab plus trastuzumab in combination with standard neoadjuvant anthracycline-containing and anthracycline-free chemotherapy regimens in patients with HER2-positive early breast cancer: a randomized phase II cardiac safety study (TRYPHAENA). Ann Oncol 24:2278–2284 23704196 10.1093/annonc/mdt182

[8] Schmid P, Cortes J, Pusztai L, McArthur H, Kümmel S, Bergh J et al (2020) Pembrolizumab for early triple-negative breast cancer. N Engl J Med 382:810–821 32101663 10.1056/NEJMoa1910549

[9] Early Breast Cancer Trialists’ Collaborative Group (EBCTCG) (2018) Long-term outcomes for neoadjuvant versus adjuvant chemotherapy in early breast cancer: meta-analysis of individual patient data from ten randomised trials. Lancet Oncol. 19:27–39 29242041 10.1016/S1470-2045(17)30777-5 PMC5757427

[10] Sherman RE, Anderson SA, Dal Pan GJ, Gray GW, Gross T, Hunter NL et al (2016) Real-world evidence - What is it and what can it tell us? N Engl J Med 375:2293–2297 27959688 10.1056/NEJMsb1609216

[11] Petracci F, Ghai C, Pangilinan A, Suarez LA, Uehara R, Ghosn M (2021) Use of real-world evidence for oncology clinical decision making in emerging economies. Future Oncol 17:2951–2960 34044583 10.2217/fon-2021-0425

[12] Gown AM (2008) Current issues in ER and HER2 testing by IHC in breast cancer. Mod Pathol 21(Suppl 2):S8-15 18437174 10.1038/modpathol.2008.34

[13] Tolaney SM, Garrett-Mayer E, White J, Blinder VS, Foster JC, Amiri-Kordestani L et al (2021) Updated standardized definitions for efficacy end points (STEEP) in adjuvant breast cancer clinical trials: STEEP version 2.0. J Clin Oncol. 39:2720–31 34003702 10.1200/JCO.20.03613 PMC10166345

[14] Litton JK, Regan MM, Pusztai L, Rugo HS, Tolaney SM, Garrett-Mayer E et al (2023) Standardized definitions for efficacy end points in neoadjuvant breast cancer clinical trials: NeoSTEEP. J Clin Oncol 41:4433–4442 37433103 10.1200/JCO.23.00435 PMC10522109

[15] Sharma P (2018) Update on the treatment of early-stage triple-negative breast cancer. Curr Treat Options Oncol 19:22 29656345 10.1007/s11864-018-0539-8

[16] Brenton JD, Carey LA, Ahmed AA, Caldas C (2005) Molecular classification and molecular forecasting of breast cancer: ready for clinical application? J Clin Oncol 23:7350–7360 16145060 10.1200/JCO.2005.03.3845

[17] Lara-Medina F, Pérez-Sánchez V, Saavedra-Pérez D, Blake-Cerda M, Arce C, Motola-Kuba D et al (2011) Triple-negative breast cancer in Hispanic patients: high prevalence, poor prognosis, and association with menopausal status, body mass index, and parity. Cancer 117:3658–3669 21387260 10.1002/cncr.25961

[18] De-la-Cruz-Ku G, Luyo M, Morante Z, Enriquez D, Möller MG, Chambergo-Michilot D et al (2020) Triple-negative breast cancer in Peru: 2000 patients and 15 years of experience. PLoS ONE 15:e0237811 32833983 10.1371/journal.pone.0237811 PMC7444821

[19] Huang M, O’Shaughnessy J, Zhao J, Haiderali A, Cortés J, Ramsey SD et al (2020) Association of pathologic complete response with long-term survival outcomes in triple-negative breast cancer: a meta-analysis. Cancer Res 80:5427–5434 32928917 10.1158/0008-5472.CAN-20-1792

[20] Schmid P, Cortes J, Dent R, McArthur H, Pusztai L, Kümmel S et al (2024) Overall survival with pembrolizumab in early-stage triple-negative breast cancer. N Engl J Med 391:1981–1991 39282906 10.1056/NEJMoa2409932

[21] Sikov WM, Berry DA, Perou CM, Singh B, Cirrincione CT, Tolaney SM et al (2015) Impact of the addition of carboplatin and/or bevacizumab to neoadjuvant once-per-week paclitaxel followed by dose-dense doxorubicin and cyclophosphamide on pathologic complete response rates in stage II to III triple-negative breast cancer: CALGB 40603 (Alliance). J Clin Oncol 33:13–21 25092775 10.1200/JCO.2014.57.0572 PMC4268249

[22] Fisher CS, Ma CX, Gillanders WE, Aft RL, Eberlein TJ, Gao F et al (2012) Neoadjuvant chemotherapy is associated with improved survival compared with adjuvant chemotherapy in patients with triple-negative breast cancer only after complete pathologic response. Ann Surg Oncol 19:253–258 21725686 10.1245/s10434-011-1877-y PMC3892697

[23] Huang K, Jakub J, Gabriel E, Moreno-Aspitia A, McLaughlin S (2023) Overall survival following neoadjuvant chemotherapy versus adjuvant chemotherapy in clinically node negative T1 triple negative breast cancer. Ann Surg Oncol 30:7026–7035 37490162 10.1245/s10434-023-13977-4

[24] Philipovskiy A, Corral J, Dwivedi KA, Heydarian R, Gaur S (2019) Efficacy of neoadjuvant versus adjuvant chemotherapy in hispanic/latino (H/L) women with local or locally advanced triple-negative breast cancer (TNBC). In Vivo 33:1227–1234 31280213 10.21873/invivo.11594 PMC6689343

[25] Gonçalves H, Guerra MR, Duarte Cintra JR, Fayer VA, Brum IV, Bustamante Teixeira MT (2018) Survival study of triple-negative and non-triple-negative breast cancer in a Brazilian Cohort. Clin Med Insights Oncol 12:1179554918790563 30083066 10.1177/1179554918790563 PMC6071162

[26] Acevedo F, Petric M, Walbaum B, Robin J, Legorburu L, Murature G et al (2021) Better overall survival in patients who achieve pathological complete response after neoadjuvant chemotherapy for breast cancer in a Chilean public hospital. Ecancermedicalscience 15:1185 33777178 10.3332/ecancer.2021.1185 PMC7987490

[27] Antonini M, Mattar A, Bauk Richter FG, Pannain GD, Teixeira MD, Amorim AG et al (2023) Real-world evidence of neoadjuvant chemotherapy for breast cancer treatment in a Brazilian multicenter cohort: correlation of pathological complete response with overall survival. Breast 72:103577 37722319 10.1016/j.breast.2023.103577 PMC10509346

[28] Dabbagh N, Riazi H, Khayamzadeh M, Negahi A, Akbari M, Akbari ME (2022) The effect of neoadjuvant vs adjuvant chemotherapy on final outcome of patients with triple negative breast cancer. Med J Islam Repub Iran 36:61 36128267 10.47176/mjiri.36.61 PMC9448446

[29] Ayettey Anie H, Yarney J, Sanuade O, Awasthi S, Ndanu TA, Parekh AD et al (2021) Neoadjuvant or adjuvant chemotherapy for breast cancer in Sub-Saharan Africa: a retrospective analysis of recurrence and survival in women treated for breast cancer at the Korle Bu teaching hospital in Ghana. JCO Glob Oncol 7:965–975 34156868 10.1200/GO.20.00664 PMC8457842

[30] Bagegni NA, Tao Y, Ademuyiwa FO (2019) Clinical outcomes with neoadjuvant versus adjuvant chemotherapy for triple negative breast cancer: a report from the national cancer database. PLoS ONE 14:e0222358 31536530 10.1371/journal.pone.0222358 PMC6752843

[31] Schuster T, Lowe WK, Platt RW (2016) Propensity score model overfitting led to inflated variance of estimated odds ratios. J Clin Epidemiol 80:97–106 27498378 10.1016/j.jclinepi.2016.05.017 PMC5756087

[32] Dent R, Trudeau M, Pritchard KI, Hanna WM, Kahn HK, Sawka CA et al (2007) Triple-negative breast cancer: clinical features and patterns of recurrence. Clin Cancer Res 13:4429–4434 17671126 10.1158/1078-0432.CCR-06-3045

