# Supplementary material for: Effect of chemotherapy timing in triple-negative breast cancer: a real-world evidence study
Source: Breast Cancer Res Treat. 2025 May 21;212(2):225–36. doi: 10.1007/s10549-025-07716-4 (PMC12133934; doi:10.1007/s10549-025-07716-4)
Supplement: Supplementary file 1 — Supplementary file1 (PDF 159 KB) [file 10549_2025_7716_MOESM1_ESM.pdf]

SUPPLEMENTARY INFORMATION

Article title: ***Effect of Chemotherapy Timing in Triple-Negative Breast Cancer: A Real-World Evidence Study***

Journal: ***Breast Cancer Research and Treatment***

Authors:

Noiver Graciano, Lucelly López, Carlos A. Rodriguez*, Katherine Montoya, Diego M. González, Luis Rodolfo Gómez, Maycos L. Zapata, Javier Cortés

*Corresponding author: Professor, Department of Pharmacology and Toxicology, Faculty of Medicine, Universidad de Antioquia, Medellín, Colombia. E-mail: andres.rodriguez@udea.edu.co.

Table S1. Overall Survival (OS) and Event-Free Survival (EFS) in TNBC Patients Treated with NACT versus ACT in a PS-Matched Cohort using Cox Proportional Hazards Analysis, including carboplatin (CBP) as a covariate

Overall survival (OS)	HR	95% CI	P value
NACT vs ACT	0.56	0.070-4.417	0.578
Stage II vs Stage I	1.428	0.717-2.843	0.31
Stage III vs Stage I	4.435	1.741-1.130	0.0018
HG2 vs HG1	1.389	0.452-4.269	0.567
HG3 vs HG1	1.679	0.595-4.741	0.327
Age 35-50y vs <35y	1.317	0.503-3.446	0.575
Age 51-70y vs <35y	1.655	0.642-4.267	0.298
Age >70y vs <35y	2.098	0.729-6.035	0.17
No RT vs RT	1.862	1.260-2.752	0.0018
<3 CT vs ≥3 CT	1.543	0.753-3.163	0.236
Non-Anthracyclines vs Anthracyclines	0.807	0.457-1.426	0.461
**Carboplatin (CBP)**	**0.875**	**0.354-2.160**	**0.772**
Moment_NACT* Stage II	2.199	0.256-1.887	0.472
Moment_NACT* Stage III	1.449	0.156-1.348	0.744
**Event-free survival (EFS)**	**HR**	**95% CI**	**P value**
NACT vs ACT	0.85	0.185-3.878	0.831
Stage II vs Stage I	1.584	0.820-3.061	0.171
Stage III vs Stage I	4.955	1.997-1.230	0.0006
HG2 vs HG1	1.295	0.423-3.965	0.651
HG3 vs HG1	1.931	0.691-5.395	0.209
Age 35-50y vs <35y	1.301	0.539-3.139	0.558
Age 51-70y vs <35y	1.701	0.715-4.047	0.23
Age >70y vs <35y	1.824	0.676-4.922	0.236
No RT vs RT	1.774	1.221-2.577	0.0026
<3 CT vs ≥3 CT	1.307	0.650-2.628	0.453
Non-Anthracyclines vs Anthracyclines	0.698	0.401-1.213	0.202
**Carboplatin (CBP)**	**1.36**	**0.593-3.116**	**0.468**
Moment_NACT* Stage II	1.163	0.233-5.801	0.854
Moment_NACT* Stage III	0.767	0.138-4.252	0.762

Figure S1. Stratified analysis of the impact on overall survival (OS) of NACT with and without CPB vs ACT

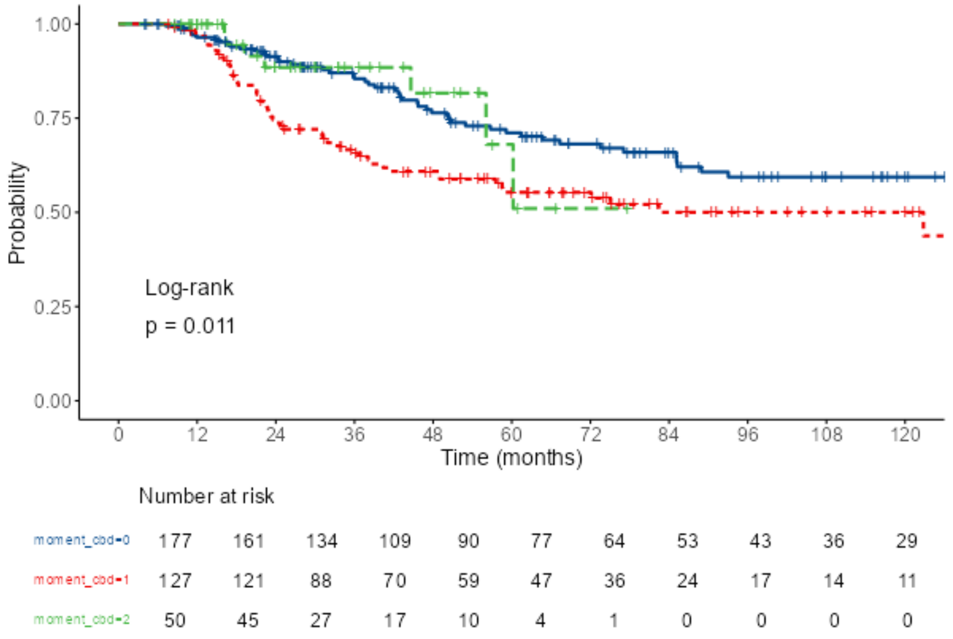

ACT: blue line, NACT without CBP: red line, NACT with CBP: green line.

Cox Table

**Levels**	**N (%)**	**HR (Univariate)**
ACT	177 (50)	-
NACT without CBP	127 (35.9)	1.7 (1.17-2.48), p=0.005
NACT with CBP	50 (14.1)	0.90 (0.41-2.00), p=0.794

Figure S2. Stratified analysis of the impact on event-free survival (EFS) of NACT with and without CPB vs ACT

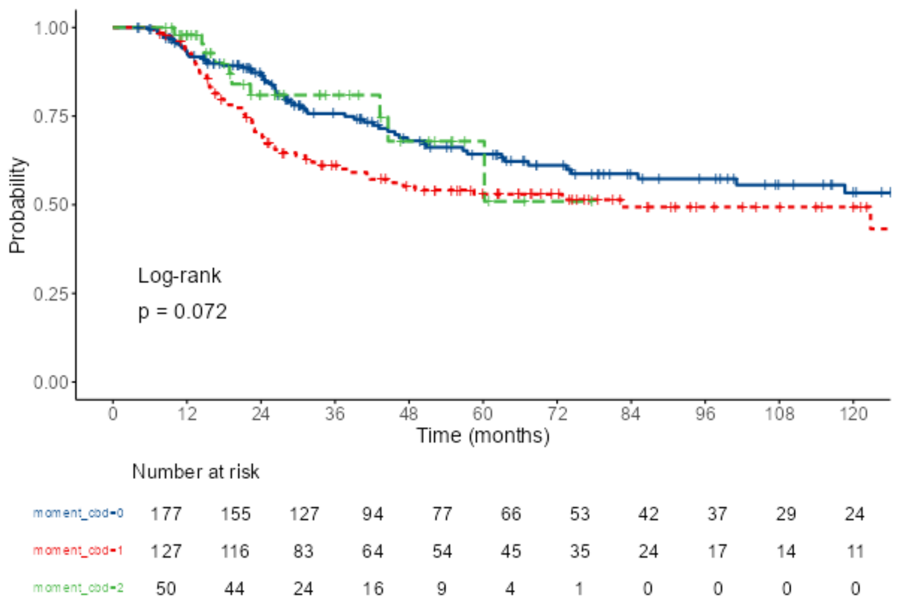

ACT: blue line, NACT without CBP: red line, NACT with CBP: green line

Cox Table

**Levels**	**N (%)**	**HR (Univariate)**
ACT	177 (50)	-
NACT without CBP	127 (35.9)	1.47 (1.03-2.11, p=0.034)
NACT with CBP	50 (14.1)	0.93 (0.47-1.82, p=0.824)
